# Observing walking with asymmetric treadmill belt speeds induces stronger activation of the action observation network than normal walking

**DOI:** 10.3389/fnhum.2025.1667742

**Published:** 2025-11-28

**Authors:** Masaya Kitamura, Tadao Ishikura, Kiyotaka Kamibayashi

**Affiliations:** 1 Graduate School of Health and Sports Science, Doshisha University, Kyoto, Japan; 2 Research Fellow of the Japan Society for the Promotion of Science, Tokyo, Japan; 3 Faculty of Health and Sports Science, Doshisha University, Kyoto, Japan

**Keywords:** action observation, functional magnetic resonance imaging, action observation network, split-belt walking, motor experience, visual familiarity

## Abstract

**Introduction:**

Observing the actions of others activates the action observation network (AON). Although previous studies have reported that motor experience and visual familiarity with an observed action can modulate the AON activity, the response of the AON to the observation of unusual walking patterns remains unclear. Therefore, this study aimed to investigate the brain activity induced by observing walking in a split-belt condition, where the left and right treadmill belt speeds differ.

**Methods:**

We examined the brain activity during the observation of video clips showing normal walking under a tied condition (the same left and right treadmill speeds) as well as walking during the initial and late periods of a split-belt condition using functional magnetic resonance imaging in 19 healthy adults. The step lengths of the actor walking in the video clips were asymmetric during the initial period of the split-belt condition and nearly symmetric during the tied condition and late period of the split-belt condition.

**Results and discussion:**

Observing the walking video clips activated broad regions of the occipito-temporo-parietal and frontal cortices, irrespective of the clip conditions. The contrasts between the conditions revealed that observing walking in the initial and late periods of the split-belt condition induced stronger activation in a subset of the AON than in the tied condition. These results suggest that observing unusual walking patterns under asymmetric speed condition induces a stronger AON activity than normal walking.

## 1 Introduction

Observing the actions of others activates motor-related brain areas (Buccino et al., 2001; Grafton et al., 1996; Hari et al., 1998; Rizzolatti et al., 1996a). This brain activity has been attributed to “mirror neurons,” first discovered in the F5 area of monkeys’ premotor cortex (di Pellegrino et al., 1992; Gallese et al., 1996; Rizzolatti et al., 1996b). Mirror neurons are characterized by firing both when a monkey executes a movement and when it observes the same movement executed by another monkey or an experimenter. To date, mirror neurons have been identified in the F5 area of monkeys’ premotor cortex (di Pellegrino et al., 1992; Gallese et al., 1996; Rizzolatti et al., 1996b) and inferior parietal lobule (IPL) (Fogassi et al., 2005). Specifically, the brain network consisting of these areas is known as the mirror neuron system (MNS) (Rizzolatti and Craighero, 2004). Studies using brain imaging techniques, such as functional magnetic resonance imaging (fMRI), have suggested the existence of the MNS in humans. Previous fMRI studies have revealed that the action observation network (AON) is activated by observing the movements of others. The AON consists of various brain areas involved in motor execution and visual processing, such as the premotor, parietal, and occipital cortices (Buccino et al., 2001, 2004; Caspers et al., 2010; Cross et al., 2009, 2012; Gazzola and Keysers, 2009; Hardwick et al., 2018; Hioka et al., 2019; Iseki et al., 2008; Pellicano et al., 2021). Pellicano et al. (2021) investigated brain activity during the execution, observation, and imagination of a walking-like motor task, in which participants moved their legs to roll a cylinder. They reported that both execution and observation of the motor task commonly activated several frontoparietal areas, including the IPL. These findings indicate that observing others’ actions activates the brain areas involved in execution of those actions. Among the AON areas, the IPL, ventral premotor cortex, and caudal part of the inferior frontal gyrus (IFG) are believed to form the core components of the human MNS (Fabbri-Destro and Rizzolatti, 2008; Rizzolatti et al., 2014).

Many studies have demonstrated that the AON activity is modulated by multiple factors related to the observed action (Kemmerer, 2021). Among these factors are the motor experience (defined as having performed the action) and visual familiarity (defined as having frequently observed the action) of the observed actions. Calvo-Merino et al. (2005) reported that dancers exhibited stronger activation in the premotor cortex, parietal cortex, and superior temporal sulcus when observing dance movements they had repeatedly practiced than when observing those they had never performed. Similarly, Cross et al. (2009) investigated the effect of a 5-day training period involving physical practice or passive observation on the AON activity during dance movement observation. They reported that, after the training, the premotor cortex and IPL exhibited stronger activation when participants observed dance sequences they had repeatedly practiced and watched than when observing untrained dance sequences. These findings indicate that motor and visual experiences with observed movements enhance the AON activity.

In contrast, several studies have demonstrated that the AON is also strongly activated by the observation of unfamiliar movements. For example, observing unpracticed guitar chords has been reported to induce stronger activation in the MNS areas, including the IPL and ventral premotor cortex, than observing practiced guitar chords (Vogt et al., 2007). Cross et al. (2012) also demonstrated that observing rigid robot-like dance movements activates the inferior parietal, premotor, and occipitotemporal cortices more strongly than observing natural human-like dance movements. These findings suggest that the AON can also be strongly activated by observing movements that observers are visually unfamiliar with or have not experienced. As mentioned above, no consensus currently exists regarding the influence of motor and visual experiences with observed movements on the AON activity during action observation.

Previous studies have investigated the effects of motor and visual experiences at various levels on the AON activity during action observation, including changes induced by short-term practice (from 1 day to several days) (Cross et al., 2009; Kirsch and Cross, 2015; Vogt et al., 2007) and comparisons between experts (athletes or musicians) and novices (Calvo-Merino et al., 2005; Haslinger et al., 2005; Kim et al., 2011; Pilgramm et al., 2010). However, these studies did not examine the daily actions that individuals perform or observe repeatedly over a long period. Therefore, the present study aimed to investigate how motor and visual experiences affect the AON activity during the observation of walking—an action performed and observed daily since childhood—by comparing brain activities during the observation of normal and unusual walking patterns. To this end, we investigated the brain activity while the participants observed video clips of normal walking in a tied condition (with the same left and right belt speeds) and walking in a split-belt condition (with different left and right belt speeds). The split-belt condition was unfamiliar to the participants as they had neither experienced nor observed it previously. When humans are exposed to the split-belt condition, their gait is initially asymmetric but gradually approaches symmetry over several minutes (Choi et al., 2009; Malone et al., 2011; Reisman et al., 2005, 2007). Therefore, we measured and compared the brain activity during the observation of walking in the initial and late periods of the split-belt condition. This comparison allowed us to examine whether perceiving the differences in the lower limb movements of others by adaptation to the walking condition affects the AON responses, even if the walking condition (i.e., the split belt) remains unchanged.

Recently, we examined changes in corticospinal excitability during the observation of other individuals walking under tied and split-belt conditions using transcranial magnetic stimulation (TMS) (Kitamura et al., 2025). We found that the corticospinal excitability of the tibialis anterior muscle was facilitated by observing symmetric walking in the tied condition and late period of the split-belt condition but not by observing asymmetric walking in the initial period of the split-belt condition. Based on these results, we speculated that the MNS is more responsive to symmetric walking, which is routinely performed and observed, than to unfamiliar asymmetric walking. However, contrasting findings have also been reported by Zhang et al. (2019). They found that during observation of race walking, elite race walkers showed reduced activity in brain areas including the IFG pars triangularis (IFGtri), premotor cortex, and supplementary motor cortex compared to novices. These findings suggest that motor expertise may enhance neural efficiency and thereby decrease AON activity. Considering these findings, motor and visual experience with observed movements may affect the AON activity during walking observation; however, this modulation might depend on the degree of the difference between observed walking patterns and those familiar to the observers. In particular, when observing walking patterns that are highly dissimilar from normal walking, such as asymmetric walking in the initial period of the split-belt condition, the AON may be less activated. Accordingly, we hypothesized that observing symmetric walking in the tied condition and late period of the split-belt condition would elicit stronger activation in motor-related brain areas than observing asymmetric walking in the initial period of the split-belt condition.

## 2 Materials and methods

### 2.1 Participants

Nineteen healthy participants with no history of neurological or psychiatric disorder (mean age ± standard deviation: 21.2 ± 1.2 years, five females) participated in this study. None of them had prior walking experience in the split-belt condition. The sample size of the present study was determined based on previous studies (Cross et al., 2009, *n* = 17; Iseki et al., 2008, *n* = 16). The study protocol was approved by the Doshisha University Research Ethics Review Committee for Human Subject Research (approval no: 23018-2) and was conducted in accordance with the principles of the Declaration of Helsinki. Written informed consent was obtained from all participants.

### 2.2 Video recording of walking and the step length of an actor

**Figure 1:**
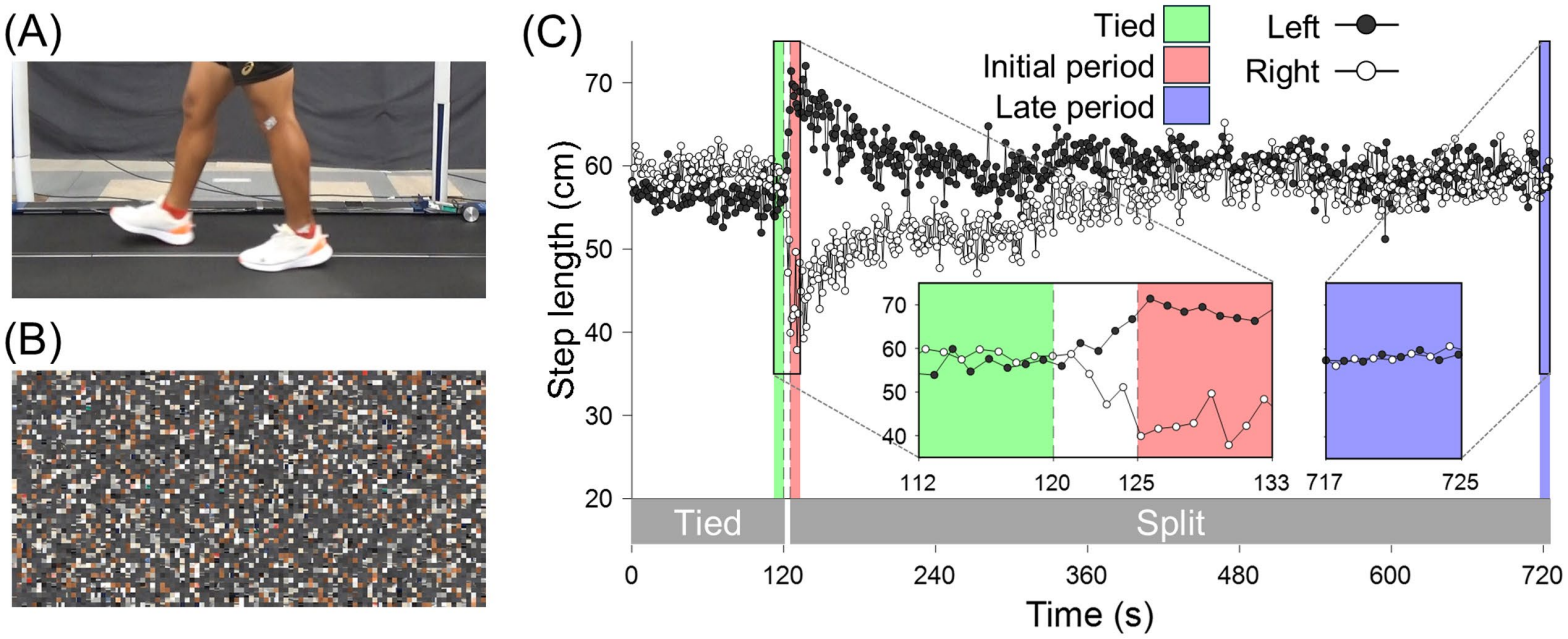
Snapshots from the walking **(A)** and scrambled video **(B)** clips. **(C)** Time series changes in the actor’s step lengths from the beginning of the tied condition at 1.25 m/s to the end of the split-belt condition. The black and white plots represent left and right step lengths, respectively. Additionally, the vertical dashed lines at 120 and 125 s indicate the end of the tied condition and the beginning of the split-belt condition, respectively. The green, red, and blue areas represent the periods shown in the video clips for the tied condition, initial period of the split-belt condition, and late period of the split-belt condition, respectively.

The walking of a male actor on a treadmill was recorded from his left side using a digital video camera (HDR-CX680, SONY, Japan) at 29.97 fps in NTSC color mode. He had no prior walking experience in the split-belt condition. Walking was performed on a treadmill (HPT-1980D-DU, Tec Gihan Co., Ltd., Japan) equipped with independently controlled left and right belts driven by separate motors. First, the actor walked under a tied condition, with the same left and right belt speeds, at 1.0, 1.5, and 1.25 m/s for 2 min each. Subsequently, he walked for 10 min in the split-belt condition, with the left and right belt speeds set at 1.0 and 1.5 m/s, respectively. The belt speed adjustments required 5 s for completion. Notably, the walking video was identical to that used in our previous study (Kitamura et al., 2025). From the recorded walking video, clips of the actor’s lower limb movements (Figure 1A) were extracted from the last 8 s of the tied condition at 1.25 m/s (tied) and from both the initial and last 8 s of the split-belt condition (initial and late periods, respectively) with a resolution of 960 × 480 pixels. Each clip contained approximately seven gait cycles. Scrambled versions of each walking clip (Figure 1B) were created as a control condition for low-level visual perception (Iseki et al., 2008). Specifically, the scrambled video clips were generated by dividing each original walking clip into 96 × 48 grids (10 × 10 pixels per grid) and randomly reordering them spatially. Since the reordered pixel positions were fixed throughout each video clip, the color changes of individual pixels and the mean luminance of each frame were identical to those in the original walking clips.

The kinematic data of the actor while walking were recorded using a motion capture system (OptiTrack, NaturalPoint Inc., USA) with a sampling rate of 100 Hz. Infrared reflective markers were placed on the bilateral ankles (lateral malleolus), and the three-dimensional position data of the markers were low-pass filtered at 6 Hz. Heel contact timing was identified as the time of peak anterior ankle position in a gait cycle, and the step length was calculated as the anteroposterior distance between the bilateral ankle markers at heel contact. The left and right step lengths were defined as the step lengths at left and right heel contacts, respectively. Figure 1C shows the time series changes in step length from the beginning of the tied condition at 1.25 m/s to the end of the split-belt condition. The left and right step lengths were almost symmetric under the tied condition. However, they became largely asymmetric during the initial period of the split-belt condition and gradually approached symmetry over several minutes.

### 2.3 Experimental procedure

**Figure 2:**
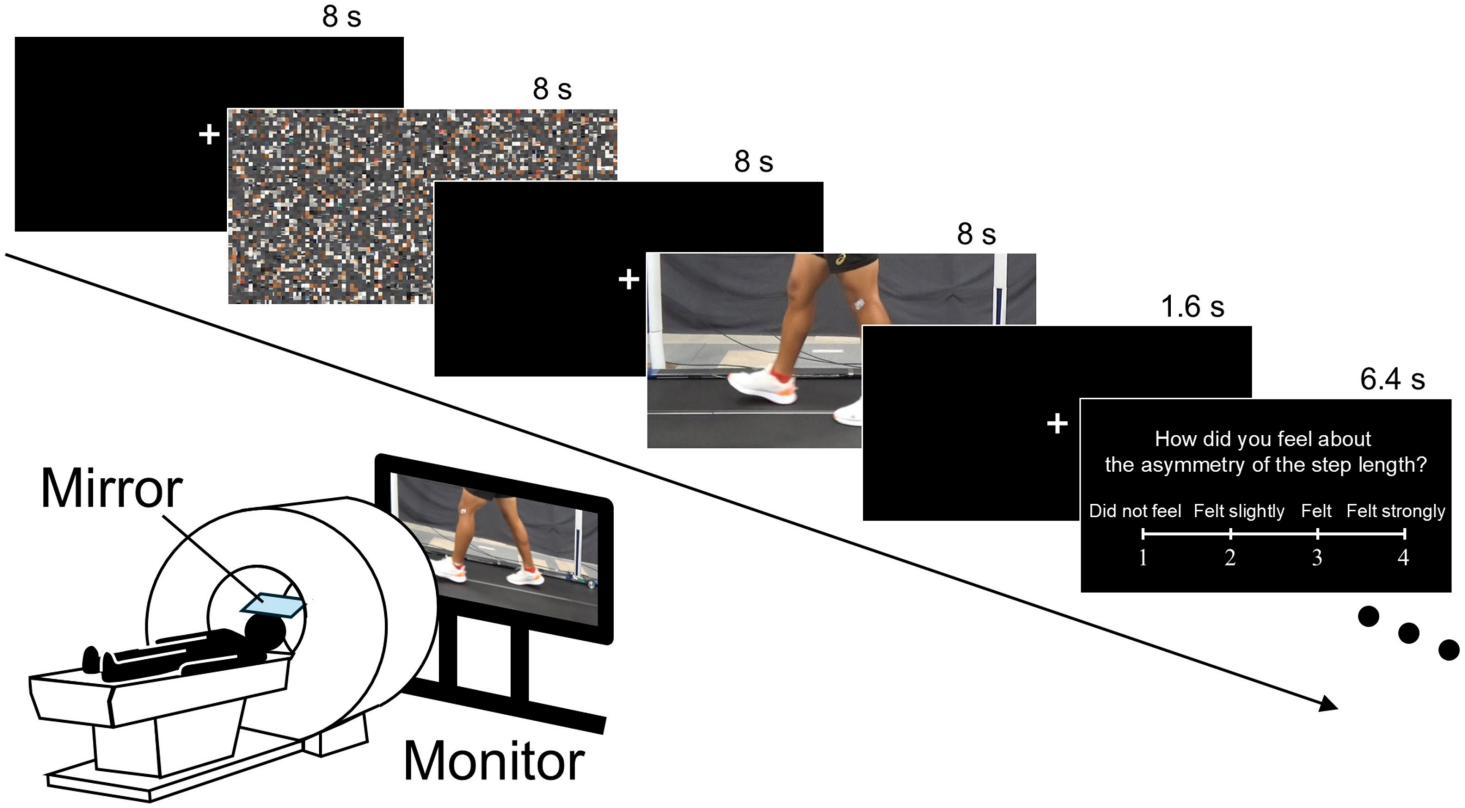
Experimental setup and time course of the fMRI experimental run. Participants observed video clips in the scanner through a mirror mounted on the head coil. An 8-s fixation cross on a black screen and an 8-s video clip were alternately presented. Occasionally, the question regarding the step length asymmetry of the observed gait appeared after observing a walking clip, and participants responded to the question using a four-point scale by pressing the response button. In this figure, the question and response scales are shown in English, while they were presented in Japanese during the actual experiment. fMRI, functional magnetic resonance imaging.

Participants observed the video clips in a 3 T MRI scanner (MAGNETOM Skyra, Siemens Healthineers, Germany) through a mirror mounted on the head coil. The video clips were presented on a monitor placed behind the scanner. Participants completed four experimental runs. In each run, four types of video clips (tied, initial period, late period, and scrambled) were each presented four times in a randomized order. In total, each type of video clip was presented 16 times throughout the experiment. Regarding the scrambled walking clips, the scrambled versions of the tied, initial period, and late period conditions were presented six, five, and five times, respectively. Each run began with a 12-s black screen, followed by alternating 8-s presentations of a white fixation cross on a black screen and 8-s video clip presentations (Figure 2). The following question about the step length asymmetry of the observed gait was occasionally presented on the monitor after observing a walking clip to evaluate how the participants perceived the asymmetry of gait in the walking clips: “How did you feel about the asymmetry of the step length?” A four-point scale was displayed along with the question as follows: 1 = “Did not feel,” 2 = “Felt slightly,” 3 = “Felt,” and 4 = “Felt strongly.” After observing the walking clip and a subsequent 1.6-s fixation cross, the question was presented for 6.4 s. Participants rated the step length asymmetry of the observed walking clip by pressing a response button (HHSC-1 × 4-L, Current Designs, USA) with their fingers based on the scale. After the question, an 8 s fixation cross was displayed before the next video clip presentation. The question was presented three times per run, and four responses were acquired for each walking clip (tied, initial period, and late period) across the four experimental runs. Timing control for the video clip presentation and the collection of participants’ responses were conducted using experimental software (Presentation Version 24.1, Neurobehavioral Systems, USA). Each experimental run lasted for 300 s. When technical errors occurred, or large body movements of the participant were observed, data from that run were discarded, and an additional run was performed. Figure 2 shows the time course of the experimental runs.

### 2.4 fMRI data acquisition

MRI images were acquired using a 3 T MRI Scanner (Siemens Healthineers) with a 32-channel head coil. Functional T2*-weighted images were collected with a multi-band echo planar imaging sequence using the following parameters: repetition time (TR) = 800 ms, echo time (TE) = 34 ms, flip angle = 90°, 66 axial slices, field of view (FoV) = 216 × 216 mm, matrix size = 86 × 86, slice thickness = 2.4 mm, interleaved order, and multi-band factor = 6. The first 15 volumes were discarded due to unstable magnetization. After completing the four experimental runs, a T1-weighted anatomical image was acquired using a magnetization-prepared rapid gradient echo sequence (TR = 2,500 ms, TE = 2.18 ms, flip angle = 8°, 224 sagittal slices, FoV = 256 × 240 mm, matrix size = 320 × 300, and slice thickness = 0.8 mm).

### 2.5 Data analysis

#### 2.5.1 Rating of the step length asymmetry

The mean rating of the step length asymmetry was calculated for each walking clip. Differences in the mean ratings across conditions were assessed using a repeated-measures one-way analysis of variance (ANOVA). Sphericity was tested using Mauchly’s test, and Greenhouse–Geisser adjustments were applied if sphericity was violated. When a significant main effect was found using ANOVA, multiple comparisons were conducted with the *p*-value adjusted using the Bonferroni method. Statistical significance was set at *p* = 0.05. The effect sizes (η_p_^2^ and Cohen’s *d*), means and 95% confidence interval (CI) of the differences between the conditions were reported. All statistical analyses were performed using IBM SPSS Statistics for Windows, version 29.0.2.0 (IBM Corp., Armonk, N.Y., USA).

#### 2.5.2 fMRI analysis

Functional and anatomical images were preprocessed using the Statistical Parametric Mapping software (SPM12, Wellcome Center for Human Neuroimaging, UK). First, functional images were realigned to correct head motion, followed by co-registration of the anatomical image to the mean functional image. Head motion was less than 2.5 mm relative to the first volume of each run. To examine whether head motion differed between conditions, we calculated framewise displacement (Power et al., 2012) during the observation of each video clip. The mean framewise displacement values were 0.15 ± 0.04, 0.16 ± 0.05, 0.16 ± 0.05, and 0.16 ± 0.04 mm for the tied, initial period, late period, and scrambled conditions, respectively. A repeated-measures ANOVA showed no significant difference between conditions (*p* = 0.073). These results suggest that walking observation did not affect head motion. Both functional and anatomical images were normalized to the Montreal Neurological Institute (MNI) space and resampled (1- and 2-mm isotropic voxels for anatomical and functional images, respectively). Normalized functional images were spatially smoothed using a Gaussian filter with a full width at half-maximum of 8 mm.

Statistical analyses were performed at two levels using SPM12. In the first-level analysis, the fMRI responses for each participant were modeled using a general linear model. The observation of the four types of video clips was modeled by specifying their start times and durations; the resulting time series were then convolved with a canonical hemodynamic response function. A high-pass filter (128 s) was applied to remove low-frequency noise, and the “FAST” temporal autocorrelation model in SPM12 was applied. Contrasts between observing the walking and scrambled video clips were calculated to identify the brain areas activated by each walking clip (tied > scrambled, initial period > scrambled, late period > scrambled). The scrambled versions of the tied, initial period, and late period conditions were treated as a single scrambled condition, regardless of the original video clip conditions. We created contrasts between the walking clip conditions (initial period > tied, late period > tied, initial period > late period, and all reverse contrasts). In the second-level group analysis, we conducted one-sample t-tests with a voxel-wise threshold of *p* = 0.001 (uncorrected) and a family-wise error (FWE) rate-corrected cluster-level threshold of *p* = 0.05 using the contrast images created in the first-level analysis. These statistical thresholds have been widely used in previous fMRI studies (Georgescu et al., 2014; Kirsch and Cross, 2015) and have been recommended based on a simulation study (Woo et al., 2014). The anatomical locations of the activated brain areas were determined using the Automated Anatomical Labeling Atlas 2 (AAL2; Rolls et al., 2015; Tzourio-Mazoyer et al., 2002). Anatomical labeling for regions included in the cluster extent was performed with the AAL2 toolbox.

## 3 Results

### 3.1 Ratings of the step length asymmetry

**Figure 3:**
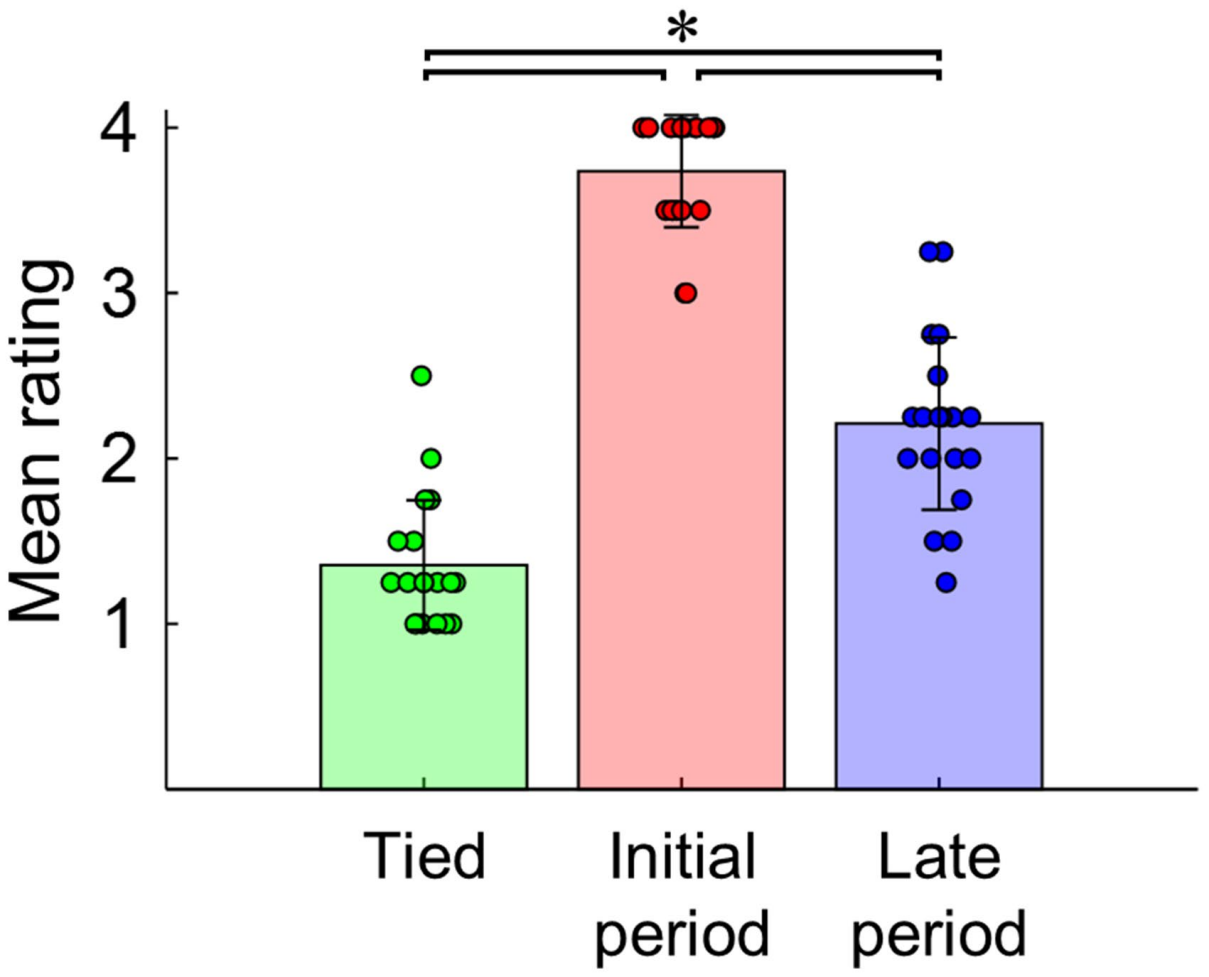
Mean ratings of the step length asymmetry for each walking clip. The bar graphs and dots represent means and individual data for each condition. The error bars indicate the standard deviations. *: *p* < 0.001.

Figure 3 shows the mean ratings of the step length asymmetry for each walking clip. The one-way ANOVA revealed a significant main effect of the condition (*F*_2,36_ = 168.765, *p* < 0.001, η_p_^2^ = 0.904). Post-hoc tests revealed that the mean rating for the initial period condition was significantly higher than that for both the tied and late period conditions (Tied-Initial period: *p* < 0.001, *d* = −4.291, mean difference = −2.382, 95% CI [−2.718, −2.045]; Initial period-Late period: *p* < 0.001, *d* = 2.900, mean difference = 1.526, 95% CI [1.208, 1.845]). Furthermore, the mean rating for the late period condition was significantly higher than that for the tied condition (*p* < 0.001, *d* = −1.356, mean difference = −0.855, 95% CI [−1.237, −0.473]).

### 3.2 Observation of the walking versus scrambled video clips

**Figure 4:**
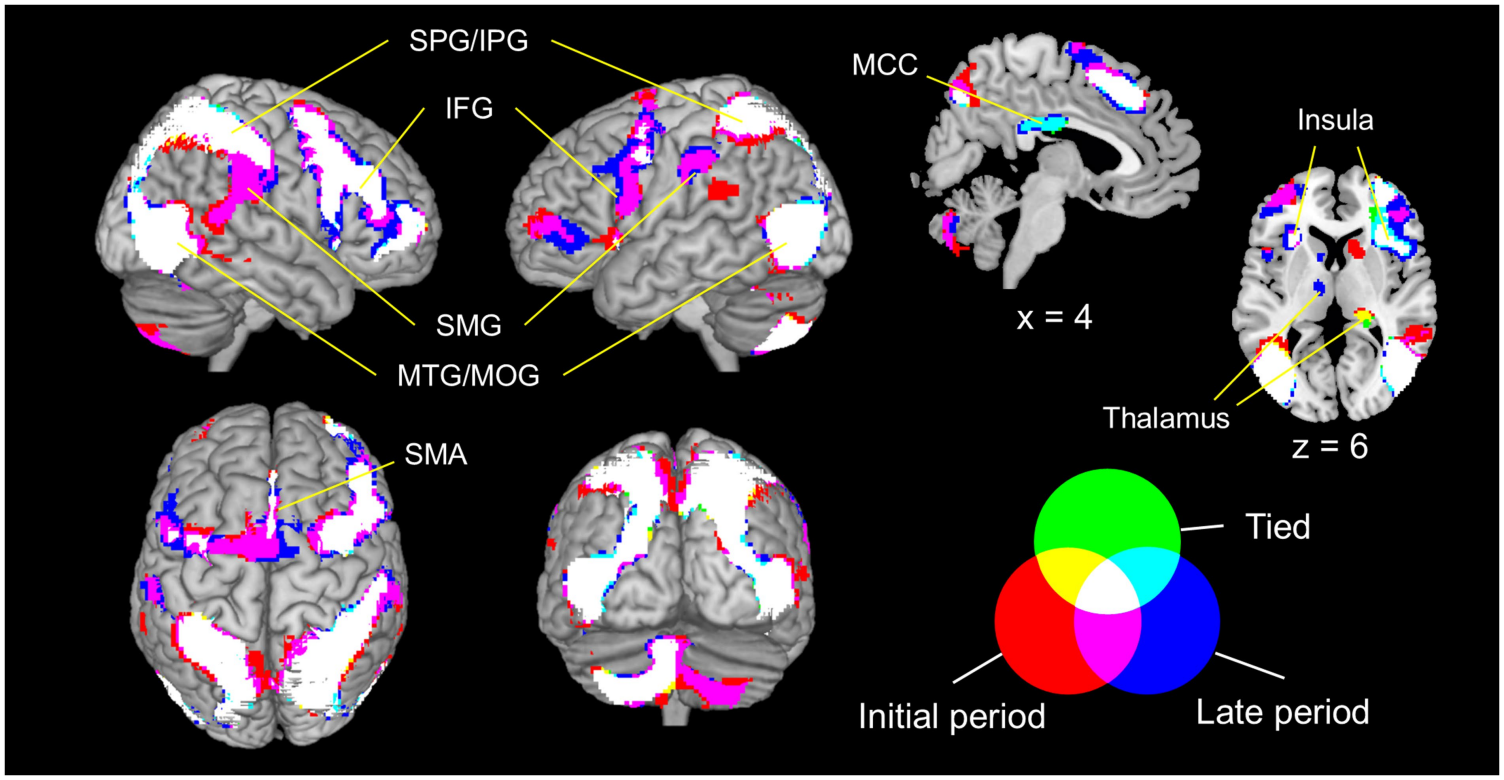
Significant clusters for the contrasts between the observation of the walking and scrambled clips (voxel level: *p* < 0.001, uncorrected; cluster level: *p* < 0.05, FWE-corrected). The green, red, and blue areas represent the clusters in each contrast (green: tied > scrambled; red: initial period > scrambled; blue: late period > scrambled). Overlapping areas are visible in mixed colors. The images are created by overlaying activation maps on a ch2better template using the MRIcron. PreCG, precentral gyrus; IFG, inferior frontal gyrus; SMA, supplementary motor area; MTG, middle temporal gyrus; SPG, superior parietal gyrus; IPG, inferior parietal gyrus; SMG, supramarginal gyrus; MOG, middle occipital gyrus; MCC, middle cingulate cortex and paracingulate gyri; FWE, family-wise error.

Figure 4 and Supplementary Table 1 present the significant clusters for the contrasts between observing the walking and scrambled clips. Irrespective of the observed walking clip conditions, common activation was found in the occipito-temporo-parietal and frontal cortices, including the middle occipital gyrus, posterior part of the middle temporal gyrus (MTG), superior and inferior parietal gyri, supplementary motor area (SMA), IFG pars opercularis (IFGoper) and IFGtri, and anterior insula (indicated in white in Figure 4). Furthermore, the left cerebellum was consistently activated.

Expansion of the activation areas was observed in many clusters under the initial and late period conditions (indicated in purple in Figure 4). The occipito-temporo-parietal activation area extended to the bilateral supramarginal gyrus (SMG) and right superior temporal gyrus. In the frontal lobe, the bilateral SMA and right IFG were consistently activated under all conditions, whereas the left IFG was further activated in the initial and late period conditions. While only the left cerebellar hemisphere was activated in the tied condition, bilateral cerebellar hemispheres were activated in the initial and late period conditions.

Several brain areas showed different activity depending on the condition. Activity in the middle cingulate/paracingulate gyri was found during the tied and late period conditions (indicated in light blue in Figure 4) but not during the initial period condition. Furthermore, activity in the subcortical areas, including the thalamus, was observed in the right hemisphere during the tied and initial period conditions, as well as in the left hemisphere during the late period condition.

### 3.3 Observation of split-belt walking versus normal walking

**Figure 5:**
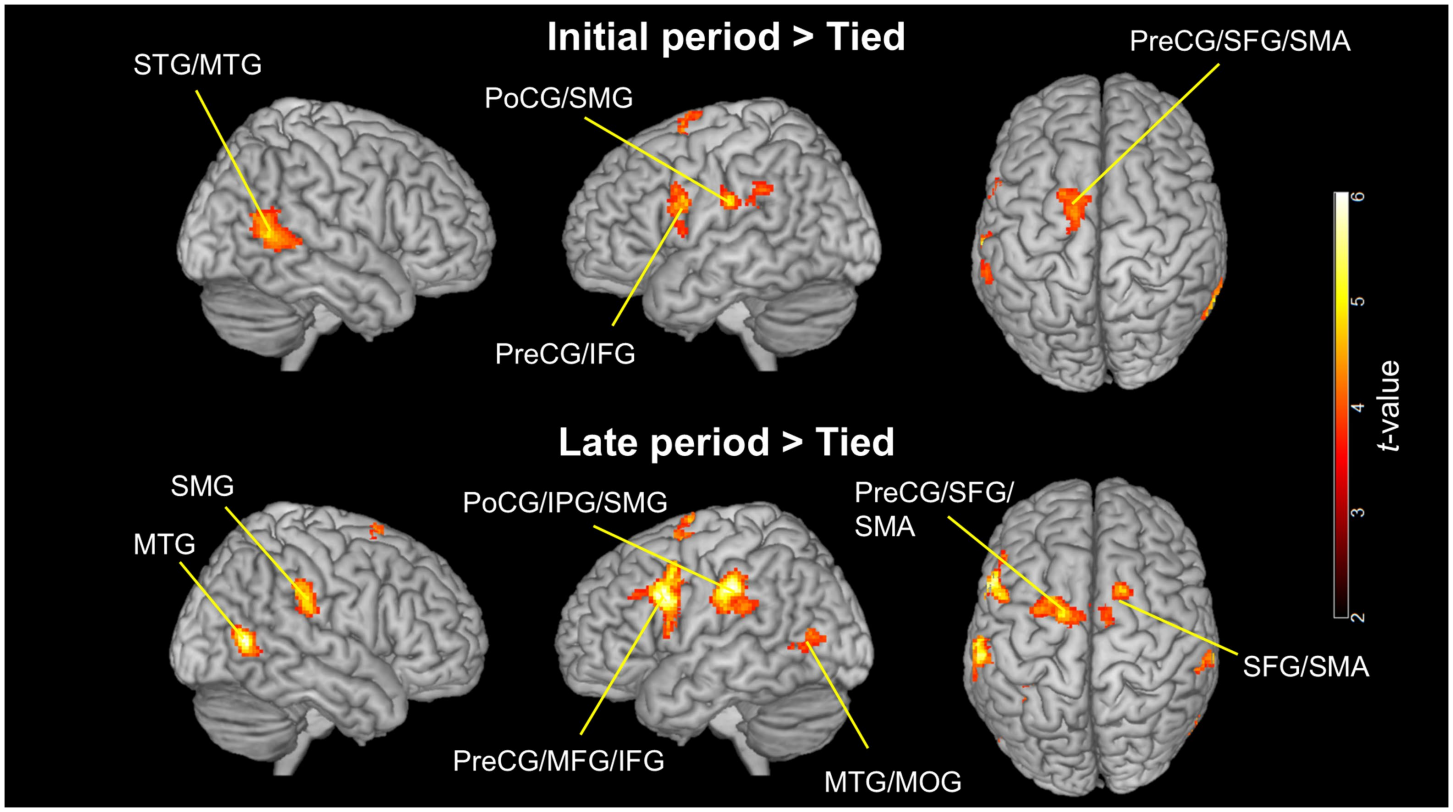
Significant clusters for the contrasts between observing the walking clips in the split-belt and tied conditions (voxel level: *p* < 0.001, uncorrected; cluster level: *p* < 0.05, FWE-corrected). The upper images show the contrasts of the initial period > tied condition, and the lower images show those of the late period > tied condition. The images are created by overlaying activation maps on a ch2better template using the MRIcron. PreCG, precentral gyrus; PoCG, postcentral gyrus; SFG, superior frontal gyrus (dorsolateral); MFG, middle frontal gyrus; IFG, inferior frontal gyrus; SMA, supplementary motor area; STG, superior temporal gyrus; MTG, middle temporal gyrus; IPG, inferior parietal gyrus; SMG, supramarginal gyrus; MOG, middle occipital gyrus; FWE, family-wise error.

Figure 5 and Table 1 present the significant clusters for the contrasts of the initial period > tied condition and late period > tied condition. These clusters contained common brain areas, including the left SMG, postcentral gyrus, precentral gyrus, IFGoper, superior frontal gyrus, SMA, and the posterior part of the right MTG. The contrast of the late period > tied condition revealed more extensive brain activations than that of the initial period > tied condition. These included additional regions, such as the left posterior MTG, middle occipital gyrus, right SMG, SMA, and superior frontal gyrus. Furthermore, the clusters in the left parietal and frontal areas were larger in the contrasts of the late period > tied condition than those of the initial period > tied condition. The parietal cluster extended into the inferior parietal gyrus, while the frontal cluster extended into the IFGtri and middle frontal gyrus. No significant clusters were found in the reverse contrasts (tied condition > initial period and tied condition > late period).

**Table 1:** Significant clusters for the contrasts of the initial period > tied condition and late period > tied condition.

Cluster extent	*p*-value	Cluster size (mm^3^)	*z*-score	MNI coordinates			Peak region
				x	y	z	
Initial period > Tied condition							
L. PoCG, SMG	0.001	2,424	4.36	−50	−28	26	L. SMG
R. STG, MTG	< 0.001	2,776	4.21	64	−54	6	R. MTG
L. PreCG, IFGoper	0.048	1,128	3.87	−58	6	26	L. PreCG
L. PreCG, SFG, SMA	0.004	1904	3.87	−16	−6	72	L. SFG
Late period > Tied condition							
L. PoCG, IPG, SMG	< 0.001	7,496	5.11	−60	−26	36	L. SMG
L. PreCG, MFG, IFGoper, IFGtri	< 0.001	5,488	4.66	−56	14	32	L. IFGoper
R. MTG	< 0.001	2,864	4.59	44	−62	8	R. MTG
L. MTG, MOG	0.018	1,464	4.43	−42	−66	10	L. MTG
R. SMG	0.001	2,688	4.24	60	−30	32	R. SMG
R. SFG, SMA	0.008	1728	4.22	16	12	64	R. SMA
L. PreCG, SFG, SMA	< 0.001	3,688	4.20	−26	−2	62	L. SFG

Anatomical regions were determined based on the AAL2. For each cluster, regions accounting for >5% of the cluster size are listed using cluster labeling in the AAL2 toolbox.

PreCG, precentral gyrus; PoCG, postcentral gyrus; SFG, superior frontal gyrus (dorsolateral); MFG, middle frontal gyrus; IFGoper, inferior frontal gyrus, (pars opercularis); IFGtri, inferior frontal gyrus (pars triangularis); SMA, supplementary motor area; STG, superior temporal gyrus; MTG, middle temporal gyrus; IPG, inferior parietal gyrus excluding the supramarginal and angular gyri; SMG, supramarginal gyrus; MOG, middle occipital gyrus; AAL2, Automated Anatomical Labeling Atlas 2.

### 3.4 Observation of walking in the initial versus late periods of the split-belt condition

**Figure 6:**
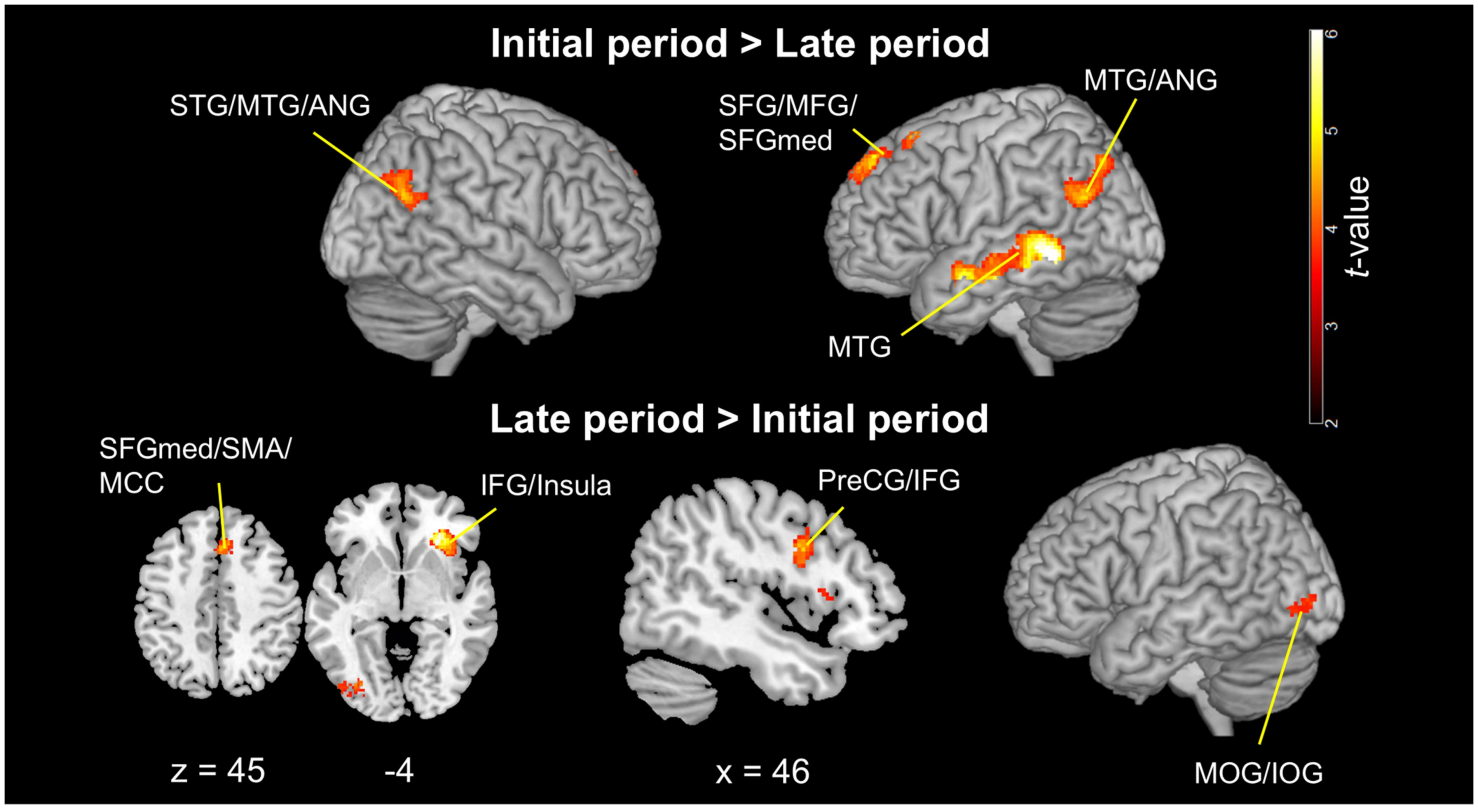
Significant clusters for the contrasts between observing the walking clips in the initial and late periods of the split-belt condition (voxel level: *p* < 0.001, uncorrected; cluster level: *p* < 0.05, FWE-corrected). The upper images show the contrasts of the initial period > late period, and the lower images show those of the late period > initial period. The images are created by overlaying activation maps on a ch2better template using the MRIcron. PreCG, precentral gyrus; SFG, superior frontal gyrus (dorsolateral); MFG, middle frontal gyrus; IFG, inferior frontal gyrus; SFGmed, superior frontal gyrus (medial); SMA, supplementary motor area; MTG, middle temporal gyrus; MOG, middle occipital gyrus; IOG, inferior occipital gyrus; MCC, middle cingulate cortex and paracingulate gyri; FWE, family-wise error.

Figure 6 and Table 2 present the significant clusters for the contrasts between observing the walking clips from the initial and late periods of the split-belt condition.

**Table 2:** Significant clusters for the contrasts of the initial > late period and late > initial period.

Cluster extent	*p*-value	Cluster size (mm^3^)	*z*-score	MNI coordinates			Peak region
				x	y	z	
Initial > Late period							
L. MTG	< 0.001	10,880	5.42	−62	−42	−2	L. MTG
R. STG, MTG, ANG	0.001	2,840	4.35	50	−60	24	R. ANG
L. MTG, ANG	< 0.001	5,912	4.30	−44	−60	22	L. MTG
L. SFG, MFG, SFGmed	< 0.001	5,232	4.12	−26	20	44	L. MFG
Late > Initial period							
R. IFGoper, IFGtri, Insula	< 0.001	4,216	4.59	28	32	−4	
L. MOG, IOG	0.022	1,648	4.00	−30	−80	0	L. MOG
L. SFGmed, SMA	0.026	1,584	3.97	12	28	30	R. MCC
R. SFGmed, SMA, MCC							
R. PreCG, IFGoper	0.020	1,680	3.84	38	8	26	R. IFGoper

Anatomical regions were determined based on the AAL2. For each cluster, regions accounting for >5% of the cluster size are listed using cluster labeling in the AAL2 toolbox. The blank indicates that the anatomical region at that MNI coordinate is not defined in the atlas.

PreCG, precentral gyrus; SFG, superior frontal gyrus (dorsolateral); MFG, middle frontal gyrus; SFGmed, superior frontal gyrus (medial); SMA, supplementary motor area; IFGoper, inferior frontal gyrus (pars opercularis); IFGtri, inferior frontal gyrus (pars triangularis); STG, superior temporal gyrus; MTG, middle temporal gyrus; ANG, angular gyrus; MCC, middle cingulate and paracingulate gyri; MOG, middle occipital gyrus; IOG, inferior occipital gyrus; AAL2, Automated Anatomical Labeling Atlas 2.

The contrast of the initial > late period revealed the following four significant clusters: two around the bilateral temporoparietal junction (TPJ), one in the left temporal lobe, and one in the left frontal lobe. The bilateral clusters around the TPJ encompassed the angular gyrus and posterior part of the MTG with the right cluster additionally involving the superior temporal gyrus. The temporal cluster covered a large area of the MTG, whereas the frontal cluster included the dorsolateral and medial parts of the superior frontal gyrus, as well as the middle frontal gyrus.

Similarly, the contrast of the late > initial period revealed four significant clusters as follows: three and one in the frontal and occipital lobes, respectively. One of the frontal clusters was located in the medial frontal cortex, encompassing the bilateral medial part of the superior frontal gyri and anterior part of the SMA (presupplementary motor area [pre-SMA]), as well as the right middle cingulate/paracingulate gyrus. The remaining two frontal clusters were located in the right hemisphere, with one consisting of the IFGoper and precentral gyrus and the other including the anterior insula, IFGtri, and IFGoper. A cluster encompassing the middle and inferior occipital gyri was observed in the left occipital lobe.

## 4 Discussion

This study aimed to investigate how motor experience and visual familiarity influence brain activity during the observation of walking in the tied condition as well as in the initial and late periods of the split-belt condition. The observers had not experienced and were visually unfamiliar with walking in the split-belt condition. Our results showed that observing other individuals’ walking activated a wide range of the occipito-temporal–parietal and frontal cortices as well as the left cerebellum, irrespective of the observed walking condition. These findings indicate that the AON is activated by walking observation. Interestingly, the contrasts between walking conditions revealed that observing the split-belt walking induced stronger activation of a subset of the AON nodes than observing normal walking in the tied condition. Changes in brain activity were found in some brain areas in the contrasts between the initial and late periods of the split-belt condition. The findings are discussed below.

The step lengths of the left and right legs in the initial period of the split-belt condition were rated as more asymmetric than those in the tied condition and late period of the split-belt condition. These results align with the actual step length asymmetry of the actor (Figure 1C), suggesting that the participants could visually perceive the gait asymmetry of the observed walking. While the step lengths of walking in the late period of the split-belt condition were perceived as more asymmetric than those in the tied condition, the actual step lengths of the actor were almost symmetric. These results align with those of our recent study (Kitamura et al., 2025). This inconsistency may be attributed to the asymmetry of gait parameters other than the step length. Previous studies have reported that step lengths gradually approach symmetry during walking in the split-belt condition (Choi et al., 2009; Reisman et al., 2005, 2007). However, some gait parameters—such as stride length (the distance traveled by ankle marker from foot contact to lift-off) and stance time—remain asymmetric throughout the exposure to the condition (Choi et al., 2009; Reisman et al., 2005, 2007). Consistent with these reports, the actor in the present study exhibited persistent asymmetry in stride length during walking in the split-belt condition (see Supplementary Data Sheet 1). Therefore, the observers might have misperceived the actor’s step lengths to be asymmetric owing to the asymmetry of the stride length or stance time.

Previous studies have reported that the MNS areas, comprising the IFG and IPL, are activated by observing normal walking (Hioka et al., 2019; Iseki et al., 2008). Consistent with these reports, we found similar activation patterns, including the IFG and inferior parietal gyrus (a part of the IPL), suggesting that the AON is activated not only by observing voluntary movements (Buccino et al., 2001, 2004; Cross et al., 2009, 2012; Gazzola and Keysers, 2009) but also by observing semi-automatic rhythmic movements such as walking. So far, studies using single-photon emission computed tomography revealed that the SMA is activated during walking execution (Fukuyama et al., 1997; Hanakawa et al., 1999). Similarly, the present study demonstrated SMA activation during walking observation, supporting the notion that action execution and observation share a partially common neural basis (Hardwick et al., 2018). Furthermore, the left cerebellum was consistently activated during walking observation. Although a recent meta-analysis did not observe cerebellar activation during action observation (Hardwick et al., 2018), a previous study indicated that the cerebellum is involved in the visual perception of biological motion (Sokolov et al., 2010). They reported that patients with tumors in the left lateral cerebellar cortex showed deficits in the visual perception of point-light displays of human walking. Therefore, the left cerebellar cortex may be involved in the visual processing of complex motor actions such as walking.

A recent TMS study (Kitamura et al., 2025) reported that the corticospinal excitability of the tibialis anterior muscle was facilitated while observing walking in the tied condition and late period of the split-belt condition, but not in the initial period of the split-belt condition. Therefore, we hypothesized that observing step-length-symmetric walking in the tied condition and late period of the split-belt condition would induce stronger activation of the AON than observing asymmetric walking in the initial period of the split-belt condition. However, the present results showed that walking observation in both periods of the split-belt condition induced stronger activation of several AON nodes than walking observation in the tied condition. In contrast, no brain area showed stronger activation during observation of walking in the tied condition.

These results may be explained by differences in the framework of neural efficiency and prediction error. In terms of neural efficiency, Zhang et al. (2019) reported that, during video observation of race walking, elite race walkers show reduced activity in brain areas, including the IFGtri, premotor cortex, and SMA, compared to novices. Their findings suggest that motor expertise with observed movements enhances neural efficiency of the AON. Similarly, Chen et al. (2020) found that baseball batters with extensive experience showed weaker activation of the AON nodes than those with intermediate experience while observing pitching actions to anticipate whether the pitch would be a strike or a ball. This pattern suggests that a greater visual/perceptual experience with specific actions leads to more efficient (i.e., less resource-intensive) neural processing in the AON. Walking is one of the most fundamental human movements, and our participants have been routinely observing it for many years. Therefore, the reduced brain activation during walking observation in the tied condition may represent the higher neural efficiency of the AON when observing familiar walking patterns compared to the unfamiliar patterns in the split-belt condition.

Conversely, the stronger AON activation during split-belt walking observation may reflect the neural processing of greater prediction errors elicited by the observation of unfamiliar walking patterns. Predictive coding theory (Kilner et al., 2007a, 2007b) posits that observers predict the kinematics of an observed movement. A comparison between the predicted and actual observed kinematics generates a prediction error. So far, it has been suggested that prediction errors affect the AON activity (Cross et al., 2012; Liew et al., 2013). Cross et al. (2012) reported that the AON areas, including the inferior parietal, premotor, and occipitotemporal cortices, were more strongly activated by observing rigid and robot-like dance movements than by observing natural and human-like dance movements. Similarly, Liew et al. (2013) showed that observing actions performed with a residual limb, which were novel to observers, induced stronger activation in areas including the bilateral IPL compared with observing familiar hand actions. These studies have suggested that a stronger AON activation, especially in the IPL, arises from prediction errors that occur when observing unfamiliar movements compared with when observing familiar, natural movements. In this context, the stronger AON activation during the split-belt walking observation is likely the neural consequence of processing such prediction errors.

During motor execution, the sensorimotor system predicts the sensory consequence of an action and continuously adjusts the action based on discrepancies between the predicted and actual sensory feedback (Wolpert et al., 2011). In contrast, during action observation, the perception of prediction errors relies solely on visual information owing to the absence of proprioceptive or vestibular feedback. Therefore, the increased activity in the posterior occipitotemporal cortices —regions involved in the visual perception of biological motion (Pelphrey et al., 2005)—during the split-belt walking observation may reflect an increased demand for visual processing to detect others’ movement errors. Moreover, the detected prediction error might be transmitted to higher-order MNS areas, such as the IFG and SMG, to update motor prediction (Kilner et al., 2007a, 2007b). The increased activity in these MNS areas may represent compensatory mechanisms that rely on visual information to update motor prediction in the absence of proprioceptive or vestibular feedback.

In the contrasts within the split-belt condition, observation of largely asymmetric walking in the initial period of the split-belt condition elicited stronger activation in the bilateral TPJ than observation of more symmetric walking in the late period. A previous study examining brain activity during the observation of computer-generated character actions reported that the left TPJ showed stronger activation when observing unfamiliar, rigid, and robot-like movements than when observing familiar and smooth movements (Georgescu et al., 2014). Therefore, the increased TPJ activity during walking observation in the initial period of the split-belt condition may reflect the processing of larger prediction errors by the visually unusual asymmetric walking pattern.

Meanwhile, stronger activation of the medial frontal cortex, encompassing the pre-SMA, was found during walking observation in the late period. A previous study has shown that neurons in the pre-SMA are strongly activated to update the motor plan (Shima et al., 1996). The gait in the late period of the split-belt condition resembles normal walking; however, because the gait was not yet completely symmetrical, prediction errors likely persisted to some extent. The increased activation of the pre-SMA may reflect the neural process related to updating internal motor representations based on small prediction errors.

Additionally, we found that walking observation in the initial period more strongly activated not only the bilateral TPJ (angular gyrus/MTG) but also a large area of the left MTG and the left frontal gyri than in the late period. Conversely, walking observation in the late period elicited stronger activation in the right anterior insula, IFG, precentral gyrus, and middle and inferior occipital gyri, as well as the medial frontal cortex. One possible explanation for these brain activity changes may be differences in visual attention required to judge step length asymmetry. The right IFG is more strongly activated during cognitive tasks with high visual search difficulty than during those with low difficulty (Mayer et al., 2007) and has also been implicated in reorienting attention during cognitive tasks (Weissman et al., 2006). The right anterior insula shows greater activation when a task becomes more challenging (Eckert et al., 2009). Furthermore, visual–spatial attention is associated with increased activity in visual cortical areas (Martínez et al., 1999). In the present study, the observers perceived the step lengths of walking in the initial period of the split-belt condition as more asymmetric than those in the late period of the split-belt condition. Therefore, while gait asymmetry in the initial period of the split-belt condition was easily identifiable, greater visual attention to the observed movements may have been required to judge gait asymmetry in the late period of the split-belt condition. This increased attentional demand could account for the increased activation of the right IFG and anterior insula, as well as the middle occipital gyrus, during walking observation in the late period. Moreover, it has been reported that the left MTG and angular gyrus are more deactivated during cognitive tasks with high visual search difficulty than those with low difficulty (Mayer et al., 2010). Considering this finding, the stronger activation of these areas during walking observation in the initial period might reflect a relative deactivation of these areas during the late period, when greater visual attention was required to judge gait asymmetry.

To date, both fMRI and TMS have been widely used to investigate how motor and visual experiences with observed actions affect central nervous system excitability during action observation (Aglioti et al., 2008; Calvo-Merino et al., 2005; Cross et al., 2009, 2012; Jola et al., 2012). However, the relationship between AON activity measured using fMRI and corticospinal excitability assessed with TMS remains unclear. Based on the findings of the present study and our previous TMS research (Kitamura et al., 2025), the results obtained from fMRI and TMS during action observations did not show corresponding changes, suggesting that the AON activity and corticospinal excitability reflect different aspects of neural processing. Although fMRI is a useful method for examining brain activity, its results cannot distinguish inhibitory and excitatory activities (Raichle, 1998). Therefore, the increased AON activity in the present study may partly reflect enhanced inhibitory neural processes and does not necessarily result in a corresponding increase in corticospinal excitability. In the present fMRI study, the contrasts between the initial and late periods of the split-belt condition showed that brain activity related to visual perception of unusual and awkward movements (e.g., TPJ) is more prominent in the initial period. Considering these findings together with our previous TMS study (Kitamura et al., 2025), which showed no increase in corticospinal excitability when observing walking in the initial period of the split-belt condition, brain activity induced by large prediction errors during excessively unusual movements might decrease corticospinal excitability.

The present study has some limitations. First, the video clips displayed a single actor from a single perspective. Changing the viewing perspective of video clips may shift observers’ attention, potentially leading to changes in brain activation patterns (e.g., lateralization). The limited diversity of video clips may have reduced the ecological validity of the findings. If the observed effects were influenced by gait characteristics specific to the actor, or by differences in idiosyncratic features of the clips (e.g., lighting, contrast) between conditions, the generalizability of the present results would be limited. Second, this study did not reveal changes in functional connectivity between brain areas. Third, we did not evaluate behavioral measures that could be linked to brain activity during action observation. Therefore, it remains uncertain whether the observed differences in brain activity between conditions represent functionally meaningful processes, such as prediction error or neural efficiency, or rather nonspecific effects related to novelty or visual attention. The fourth limitation is that we did not include visually unfamiliar non-biological movements as a control condition for novelty or attentional effects. Moreover, because we did not incorporate observation of non-locomotor movements, it is unclear whether the observed brain activity changes are specific to observation of locomotor movements. Finally, whereas head motion did not significantly differ between conditions, even small head movements can induce fMRI signal changes (Power et al., 2012; Satterthwaite et al., 2013). For example, head motion may reduce fMRI signal within brain parenchyma while increasing signal near the rim of the brain (Satterthwaite et al., 2013). Future studies should therefore address these methodological limitations by using larger and more diverse stimulus sets with multiple actors and viewpoints as well as various control stimuli, incorporating behavioral and connectivity measures to clarify the functional meaning of brain activity, and applying advanced preprocessing techniques to minimize the impact of motion artifacts. Such approaches will enhance the ecological validity, interpretability, and robustness of findings regarding AON activity during action observation.

## 5 Conclusion

This study found that a subset of the AON nodes was more strongly activated during the observation of split-belt walking, which the observers had neither experienced nor observed, than during the observation of normal walking. The present results indicate that motor experience and visual familiarity with observed movements influence brain activity during the observation of highly familiar, semi-automatic actions, such as walking, which observers have performed and observed repeatedly over many years. Therefore, these findings can deepen the understanding of the neural basis of action observation.

## Data Availability

The raw data supporting the conclusions of this article will be made available by the authors, without undue reservation.
