# Supplementary material for: Observing walking with asymmetric treadmill belt speeds induces stronger activation of the action observation network than normal walking
Source: Front Hum Neurosci. 2025 Nov 28;19:1667742. doi: 10.3389/fnhum.2025.1667742 (PMC12698651; doi:10.3389/fnhum.2025.1667742)
Supplement: Supplementary file 1 [file Data_Sheet_1.DOCX]

# Stride length of the actor

## The stride lengths of the actor were calculated based on three-dimensional position data of the markers placed on the bilateral ankles (lateral malleolus). Heel contact and toe-off timings were identified as the time of peak anterior and posterior ankle positions in a gait cycle, respectively. The stride length was defined as the anteroposterior distance between heel contact and the subsequent toe-off.

Supplementary Fig 1 shows the time series changes in stride lengths from the beginning of the tied condition at 1.25 m/s to the end of the split-belt condition. The left and right stride lengths were almost symmetric in the tied condition. However, they became asymmetric throughout the split-belt condition.

##
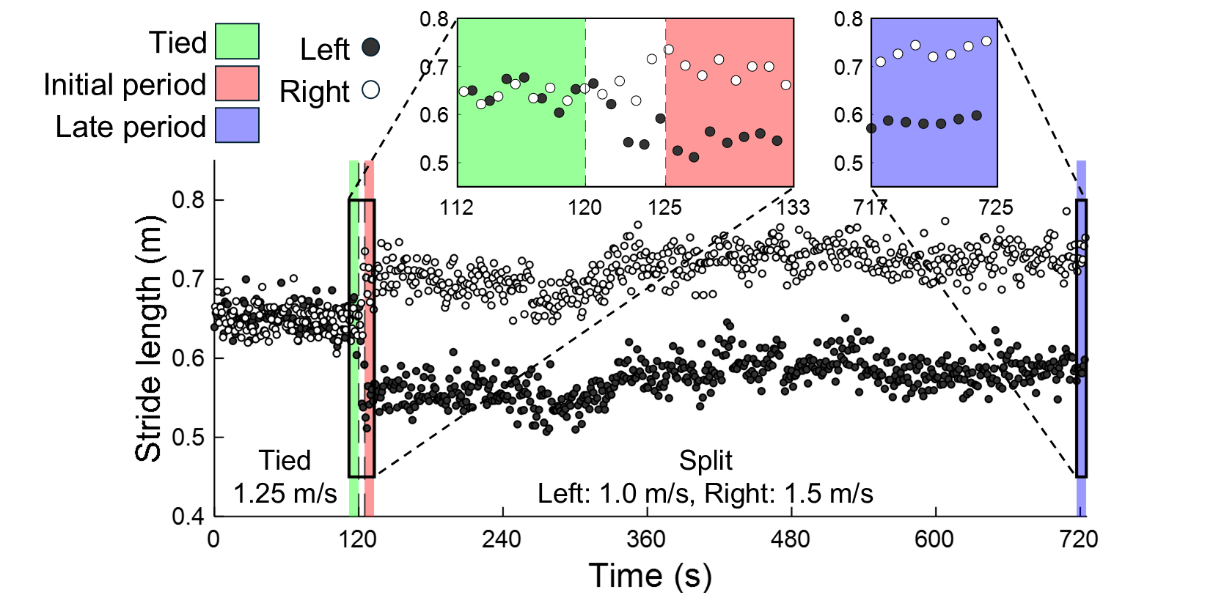


**Supplementary Figure 1.** Time series changes in the actor’s stride lengths from the beginning of the tied condition to the end of the split-belt condition. The horizontal and vertical axes represent time and stride length, respectively. The black and white dots respectively represent left and right stride lengths at in each gait cycle, plotted against the heel contact timing. The vertical dashed lines at 120 s and 125 s mark the end of the tied condition and the beginning of the split-belt condition, respectively. The green, red, and blue shaded areas correspond to the periods shown in the video clips for the tied condition, the initial period of the split-belt condition, and the late period of the split-belt condition, respectively.
