# Supplementary material for: Observing walking with asymmetric treadmill belt speeds induces stronger activation of the action observation network than normal walking
Source: Front Hum Neurosci. 2025 Nov 28;19:1667742. doi: 10.3389/fnhum.2025.1667742 (PMC12698651; doi:10.3389/fnhum.2025.1667742)
Supplement: Supplementary file 2 [file Table_1.DOCX]

Supplementary Material

# Supplementary Table

**Table S1:** Significant clusters for the contrasts between observing the walking clips and scrambled clips.

|  |  |  |  | **MNI coordinates** | | |  |
| --- | --- | --- | --- | --- | --- | --- | --- |
| **Cluster extent** | ***p*-value** | **cluster size (mm^3^)** | ***z*-score** | **x** | **y** | **z** | **Peak region** |
| ***Tied > Scrambled*** |  |  |  |  |  |  |  |
| L. PoCG, MTG, SPG, IPG, SOG, MOG | < 0.001 | 54344 | 7.10 | -48 | -76 | -2 | L. MOG |
| R. PoCG, MTG, ITG, SPG, IPG, Precuneus, SOG, MOG | < 0.001 | 69624 | 6.61 | 46 | -62 | 8 | R. MTG |
| L. PreCG, SFG, SFGmed, SMA  R. SFGmed, SMA | < 0.001 | 16632 | 5.09 | 8 | 26 | 44 | R. SFGmed |
| L. Cerebellum crus I, II, lobule VI, VIIb, VIII | < 0.001 | 13152 | 4.88 | -8 | -80 | -48 |  |
| R. PreCG, SFG, MFG, IFGoper, IFGtri, Insula | < 0.001 | 38320 | 4.86 | 30 | 36 | 8 |  |
| R. Hippocampus, Thalamus | 0.026 | 1456 | 4.80 | 14 | -30 | 14 |  |
| R. MCC | 0.005 | 2056 | 4.62 | 4 | -12 | 26 |  |
| L. Insula | 0.012 | 1728 | 4.30 | -34 | 20 | -2 | L. Insula |
| ***Initial period > Scrambled*** |  |  |  |  |  |  |  |
| L. SPG, IPG, MOG  R. MTG, SPG | < 0.001 | 154040 | 6.90 | -48 | -76 | -4 | L. IOG |
| L. Cerebellum crus I, II, lobule VI, VIIb, VIII  R. Cerebellum lobule VIIb, VIII | < 0.001 | 24456 | 6.03 | -6 | -78 | -46 |  |
| R. PreCG, SFG, MFG, IFGoper, IFGtri | < 0.001 | 40632 | 5.14 | 52 | 14 | 28 | R. IFGoper |
| L. PreCG, SFG, MFG, IFGoper, SFGmed, SMA  R. SFGmed, SMA | < 0.001 | 27672 | 4.79 | 4 | 14 | 54 | R. SMA |
| R. Hippocampus, Thalamus | 0.010 | 1768 | 4.73 | 18 | -28 | 10 | R. Thalamus |
| L. Insula | 0.004 | 2128 | 4.50 | -32 | 20 | -4 | L. Insula |
| L. SFG, MFG, IFGtri | 0.004 | 2160 | 4.36 | -32 | 52 | 6 | L. MFG |
| R. Caudate | 0.037 | 1304 | 3.91 | 16 | 12 | 2 |  |
| ***Late period > Scrambled*** |  |  |  |  |  |  |  |
| L. PoCG, MTG, SPG, IPG, Precuneus, SOG, MOG | < 0.001 | 64448 | 7.18 | -48 | -76 | -4 | L. IOG |
| R. PoCG, MTG, ITG, SPG, IPG, SMG, Precuneus, SOG, MOG | < 0.001 | 84752 | 7.02 | 46 | -62 | 8 | R. MTG |
| L. PreCG, SMA  R. SFG, MFG, IFGoper, IFGtri, SMA | < 0.001 | 100024 | 6.36 | 34 | 20 | 2 | R. Insula |
| L. Cerebellum crus I, II, lobule VI, VIIb, VIII  R. Cerebellum lobule VIIb, VIII | < 0.001 | 23416 | 6.05 | -22 | -78 | -54 |  |
| L. IFGtri, Insula | < 0.001 | 3992 | 5.71 | -30 | 24 | 8 | L. Insula |
| L. SFG, MFG, IFGtri, IFGorb | < 0.001 | 4376 | 4.75 | -46 | 48 | 6 | L. IFGtri |
| L. MCC  R. MCC | 0.001 | 2784 | 4.49 | 4 | -16 | 26 |  |
| L. Caudate, Pallidum, Thalamus | 0.012 | 1776 | 4.34 | -12 | -8 | 4 | L. Thalamus |

Anatomical regions were determined based on the AAL2. For each cluster, regions accounting for >5% of the cluster size are listed using cluster labeling in the AAL2 toolbox. The blank indicates that the anatomical region at that MNI coordinate is not defined in the atlas.

PreCG, precentral gyrus; PoCG, postcentral gyrus; SFG, superior frontal gyrus (dorsolateral); MFG, middle frontal gyrus; IFGoper, inferior frontal gyrus (pars opercularis); IFGtri, inferior frontal gyrus (pars triangularis); SFGmed, superior frontal gyrus (medial); SMA, supplementary motor area; IFGorb, inferior frontal gyrus (pars orbitalis); MTG, middle temporal gyrus; ITG, inferior temporal gyrus; SPG, superior parietal gyrus; IPG, inferior parietal gyrus excluding supramarginal and angular gyri; SMG, supramarginal gyrus; SOG, superior occipital gyrus; MOG, middle occipital gyrus; IOG, inferior occipital gyrus; MCC, middle cingulate cortex and paracingulate gyri
